# Improving the translation of search strategies using the Polyglot Search Translator: a randomized controlled trial

**DOI:** 10.5195/jmla.2020.834

**Published:** 2020-04-01

**Authors:** Justin Michael Clark, Sharon Sanders, Matthew Carter, David Honeyman, Gina Cleo, Yvonne Auld, Debbie Booth, Patrick Condron, Christine Dalais, Sarah Bateup, Bronwyn Linthwaite, Nikki May, Jo Munn, Lindy Ramsay, Kirsty Rickett, Cameron Rutter, Angela Smith, Peter Sondergeld, Margie Wallin, Mark Jones, Elaine Beller

**Affiliations:** Institute for Evidence-Based Healthcare, Bond University, Robina, Queensland, Australia, jclark@bond.edu.au, https://orcid.org/0000-0003-0133-1613; Institute for Evidence-Based Healthcare, Bond University, Robina, Queensland, Australia, ssanders@bond.edu.au; Institute for Evidence-Based Healthcare, Bond University, Robina, Queensland, Australia, macarter@bond.edu.au; Bond University Library, Bond University, Robina, Queensland, Australia, d_honeyman@hotmail.com; Institute for Evidence-Based Healthcare, Bond University, Robina, Queensland, Australia, gcleo@bond.edu.au; Gold Coast Health Library Service, Gold Coast University Hospital, Southport, Queensland, Australia, Yvonne.Auld@health.qld.gov.au; University Library, University of Newcastle, Callaghan, New South Wales, Australia, debbie.booth@newcastle.edu.au; University Library, University of Melbourne, Melbourne, Victoria, Australia, p.condron@unimelb.edu.au; University Library, University of Queensland, Brisbane, Queensland, Australia, c.dalais@library.uq.edu.au; Bond University Library, Bond University, Robina, Queensland, Australia, sbateup@bond.edu.au; Bond University Library, Bond University, Robina, Queensland, Australia, blinthwa@bond.edu.au; Sturt Library, Flinders University, Adelaide, South Australia, Australia, nikki.may@sa.gov.au; Centre for Teaching and Learning, Southern Cross University, Coffs Harbour, New South Wales, Australia, Joanne.Munn@scu.edu.au; University Library, Australian Catholic University, Banyo, Queensland, Australia, Lindy.Ramsay@acu.edu.au; University Library, University of Queensland, Brisbane, Queensland, Australia, k.rickett@library.uq.edu.au; University Library, Queensland University of Technology, Kelvin Grove, Queensland, Australia, c.rutter@qut.edu.au; Hunter New England Health Libraries, New South Wales (NSW) Health, Hunter Region, New South Wales, Australia, Angela.Smith@hnehealth.nsw.gov.au; University Library, Queensland University of Technology, Kelvin Grove, Queensland, Australia, p.sondergeld@qut.edu.au; University Library, Southern Cross University, Coffs Harbour, New South Wales, Australia, jam232@gmail.com; Institute for Evidence-Based Healthcare, Bond University, Robina, Queensland, Australia, majones@bond.edu.au; Institute for Evidence-Based Healthcare, Bond University, Robina, Queensland, Australia, ebeller@bond.edu.au

## Abstract

**Background:**

Searching for studies to include in a systematic review (SR) is a time- and labor-intensive process with searches of multiple databases recommended. To reduce the time spent translating search strings across databases, a tool called the Polyglot Search Translator (PST) was developed. The authors evaluated whether using the PST as a search translation aid reduces the time required to translate search strings without increasing errors.

**Methods:**

In a randomized trial, twenty participants were randomly allocated ten database search strings and then randomly assigned to translate five with the assistance of the PST (PST-A method) and five without the assistance of the PST (manual method). We compared the time taken to translate search strings, the number of errors made, and how close the number of references retrieved by a translated search was to the number retrieved by a reference standard translation.

**Results:**

Sixteen participants performed 174 translations using the PST-A method and 192 translations using the manual method. The mean time taken to translate a search string with the PST-A method was 31 minutes versus 45 minutes by the manual method (mean difference: 14 minutes). The mean number of errors made per translation by the PST-A method was 8.6 versus 14.6 by the manual method. Large variation in the number of references retrieved makes results for this outcome inconclusive, although the number of references retrieved by the PST-A method was closer to the reference standard translation than the manual method.

**Conclusion:**

When used to assist with translating search strings across databases, the PST can increase the speed of translation without increasing errors. Errors in search translations can still be a problem, and search specialists should be aware of this.

## BACKGROUND

Systematic reviewers are advised to search multiple electronic bibliographic databases combined with other methods to ensure all relevant studies are identified [1]. However, databases differ in terms of interfaces, field codes, thesaurus terms, and proximity operators. This means that the original search string needs to be translated multiple times into the search syntax required by each database. This process can be laborious and complex, potentially introduce errors, and increase the time spent on the systematic review (SR) search tasks [2–4].

Several groups have worked to reduce the labor and complexity of translating search strings across databases. This work has made the task easier for the groups involved, but the tools developed lack broad applicability, because they translate search strings into a limited number of databases [5] or are not easily accessed or implemented [6, 7]. These tools include Medline Transpose, which translates search strings between the Ovid MEDLINE and PubMed interfaces [5], and macros in MS Excel and Word to help with the translation of search syntax [6, 7].

The Polyglot Search Translator (PST) [8] was designed to assist with the search translation task. The PST is freely available to people needing to translate database search strings. Accessible via the Internet since 2017, the PST has been accessed over 10,000 times as of September 2019 and has received awards from Health Libraries Australia (HLA) [9, 10].

To perform a translation with the PST, users paste a PubMed or Ovid MEDLINE search string into the “Your query” box and immediately retrieve the translated search string for all the alternative databases. The translated search string should be checked and modified if necessary. In particular, Medical Subject Headings (MeSH) terms in the original search need to be replaced manually when translating to databases that do not use MeSH terminology. Users then paste the translated search string into the appropriate database and run the search. Screenshots and a description of how the version of the PST used in the trial should be used are provided in supplemental Appendix A. In this study, the authors evaluated whether the PST, when used as an aid to translate database search strings across multiple databases, reduces the time taken to perform translations without increasing translation errors.

## METHODS

We compared search string translations (from PubMed and Ovid MEDLINE to other databases) performed with the assistance of the PST (PST-A method) to translations performed without the assistance of the PST (manual method). We assessed (1) the time taken to translate the search strings, (2) the number of errors in the translated search strings, and (3) the number of references retrieved by the translated search strings, compared with the number of references retrieved by a reference standard search string translation.

### Study participants

Participants (n=20) with very limited or no experience using the PST were recruited via the Australian Library Information Association (ALIA) Health Libraries Australia (HLA) email list (n=16) and our personal contacts (n=4). The recruitment period went from September 2017 to November 2017. The trial commenced in November 2017 and ended in March 2018.

### Sample set of searches for translation

Twenty search strings were collected from published SRs, including five Cochrane intervention reviews, two drug intervention reviews, three non–drug intervention reviews, three diagnostic reviews, two prevalence reviews, two prognosis reviews, and three health technology assessments. The numbers and types of reviews were decided a priori to ensure a wide variety of search strings were used. To obtain these reviews, searches were run in PubMed and the Health Technology Assessment Database (supplemental Appendix B). SRs were randomly selected from each search by generating a random number using the Google random number generator. The SR with the search result number matching the random number was selected for further assessment. We reviewed the search string from the SR to identify those that:

- were from an SR or health technology assessment
- were in PubMed or Ovid MEDLINE format
- were provided in full the same as they were used in the database
- were in English
- included subject (MeSH) terms and keywords
- searched for some keywords in a specific field, such as the title and/or abstract
- searched for a minimum of 3 different terms
- searched for synonyms for some of the terms
- were no more than 100 lines in length

If the search string met the inclusion criteria, the search was extracted. If it did not, another random number was generated, and another SR was selected and checked against the inclusion criteria. Of the final set of twenty search strings, five were in PubMed format and fifteen were in Ovid MEDLINE format. A full list of the SRs selected to be used in the study is provided in supplemental Appendix C.

### Allocation of the search strings

Each participant was randomly allocated ten search strings from the pool of twenty. Participants who lacked access to Ovid MEDLINE and, therefore, could not translate from PubMed to Ovid MEDLINE were allocated ten from the pool of fifteen Ovid MEDLINE searches that they could translate into PubMed.

### Allocation of the translation method

Participants were randomly assigned to translate each of the ten search strings that they had been allocated by the PST-A method (five search strings) or the manual method (five search strings). Randomization was balanced so that each search string would be translated using both methods an equal number of times over all participants. The participants translated each search string from the original PubMed to Ovid MEDLINE (or vice versa) and into two other randomly selected databases. The potential databases included Embase (via Elsevier or OVID), the Cochrane Library, Cumulative Index of Nursing and Allied Health Literature (CINAHL), Web of Science, and Scopus.

We aimed to balance the number of times each string was translated into each database by each of the two methods. However, as not all participants had access to all databases, their allocations were adjusted to account for this. For example, four participants lacked access to Ovid MEDLINE, while two others lacked access to Scopus. Participants with similar database access were paired together, and translations were allocated to ensure balance across these pairings.

### Description of the intervention and comparator

Participants could seek help from any sources while conducting translations by either method. This could include referring to online help guides or consulting colleagues. The only exception was that they were asked not to consult with other participants in the trial.

For PST-A method translations, participants were asked to use the PST as they felt appropriate and to modify the translation done by the PST before running it if necessary. For manual method translations, participants were asked to translate the search string using their usual methods.

Participants were asked to translate the search strings to run as close as possible to the original. Participants were not initially provided with any background to the review question or the number of references retrieved by the original search. A single participant requested the number of references retrieved by the original searches and was provided with them. Information provided to participants about using the PST, trial conditions, and how to record results is provided in supplemental Appendix D.

### Blinding of participants and assessors

Participants could not be blinded to the translation method (PST-A or manual). Investigators assessing the translated search strings and the results of those translations were blinded to the participant who performed the translation and the translation method.

### Data collection

Participants were provided with a data collection form to record the time taken to translate and run each search string in each database and to record the number of references retrieved by each translation. Translated search strings were saved in the database or a document. At the end of the trial, participants were sent a survey asking them about their training, work, and SR experience.

### Development of the reference standard search string translations

To develop the reference standard set of search string translations, two of the authors translated the twenty search strings independently. The translations were compared, and discrepancies were resolved through discussion until a single, most correct, translation was agreed upon. New translations were created rather than attempting to use the translations from the original reviews since most reviews only provided the original search string.

### Number of errors in the search string translations

Each search string translation was marked independently by two authors (Clark and Honeyman), who were blinded to the method used. Professional judgment and the reference standard translation were used to determine errors, with any discrepancies resolved through discussion. Errors were marked leniently; for instance, translating [tiab] in PubMed to .ti,ab. in Ovid MEDLINE was not considered an error. However, field translations that were clearly not good matches (e.g., [tw] in PubMed to .tw. in Ovid MEDLINE) were considered an error.

### Types of errors in the search string translations

Each error in each translated search string was assigned to one of thirty-two different error categories (e.g., using the wrong wildcard or truncation syntax, choosing the wrong field such as only searching the title field instead of both the title and abstract). Each error was also classified as an error that impacted recall (missing relevant articles) and/or precision (increasing the number of irrelevant articles) [11]. Recall errors were prioritized; therefore, an error that could impact recall and precision was recorded as a recall error.

### Error counts in search string translations

Two error counts were recorded. The first was a count of the total errors made per search translation. For this, an error of the same type occurring multiple times within a search translation was counted each time (e.g., choosing the wrong field for thirty terms would count as thirty errors in that translation). The second was the total of unique errors per search string translation (e.g., choosing the wrong field for thirty terms would count as one error of that type in that search translation).

### Differences in the number of references retrieved by the translated search versus the number of references retrieved by the reference standard translation

For each translated search string, the number of references retrieved by the participant’s translation was recorded and compared to the number of references retrieved by the reference standard translation. The difference between these two numbers was calculated, and it was inferred that the greater the difference, the greater the search translation error. The difference in the number of references retrieved was expressed as a percentage and then categorized and scored (Table 1).

**Table 1:** Categorization and scores for the difference from the expected number of references

Deviation from expected number of references	Categorization	Categorization score
Between −50% and −100%	Larger negative deviation (likely to have missed relevant records)	−2
Between −50% and −5%	Smaller negative deviation (may have missed relevant records)	−1
Between −5% and 5%	No important deviation	0
Between 5% and 50%	Smaller positive deviation (some extra records to screen)	1
Greater than 50%	Larger positive deviation (likely to have many irrelevant records to screen)	2

The formula for calculating the difference from the expected number of references retrieved (referred to as closeness) was:

$$\text{Closeness}=100\times \frac{|\text{Hits}-{\text{Hits}}_{\text{reference}}|}{{\text{Hits}}_{\text{reference}}}$$

Thus, if a reference standard translation found 1,000 references, a participant’s translation that found 800 or 1,600 references would have a difference of −20% or +60%, respectively. The mean of these scores was calculated (referred to as the categorization score) to give an indication of the comparative performance of the 2 methods.

### Sample size

Based on our professional experience, we assumed an approximate time saving of 50% for the PST. Thus, for a study power of 80%, we needed 50 search strings translated by the PST (i.e., a total of 100 search strings). We did not adjust the sample size for clustering, as we did not have a reliable estimate of the intra-class coefficient. We were also unsure of the likely completion rate for translations; therefore, we increased the sample size considerably to allow for a conservative estimate of both these factors. Clustering was accounted for in the statistical analysis using mixed models. We obtained complete data for 364 strings (172 PST-A method, 192 manual method) and incomplete data for 5 search strings (4 PST-A method, 1 manual method).

### Search complexity

To determine if complexity of the search string affected the results, search strings were ranked in order of complexity from least (1) to most (20) complex by a consensus process between two of the authors (Clark and Honeyman). The ranking was also shared with participants and their feedback taken into consideration (supplemental Appendix E).

### Analysis

Due to participants dropping out and not completing all search string translations, the data were analyzed in two ways: (1) using a descriptive comparison using all the collected data and (2) using mixed models to account for the repeated measures study design and the lack of balance due to missing data. A linear mixed model was fitted to compare time taken for search strings translated with the PST-A method to those conducted using the manual method. Time was log-transformed prior to analysis to reduce positive skew. Similarly, a linear mixed model was fitted to compare the (log) closeness. For analysis of number of errors made, we fitted a generalized Poisson mixed model to account for the counts of number of errors made being highly variable, which ranged from 0 to 121. The search string and translation databases specified were included as covariates in the models, and interaction terms were initially included to test whether the effect of method of translation used (PST-A or manual) differed by search string or by translation databases.

## RESULTS

Of the 20 participants recruited, 4 (20%) completed no search translations, 6 (30%) completed some of the translations, and 10 (50%) completed all 10 of their translations. Of the 16 participants who were sent the survey, 15 responded. Participants primarily had a library background, a masters’ level education in library science, and were university based. Work experience was more varied, ranging from a recent graduate to a participant with more than 20 years’ experience. SR author experience was also mixed, with 5 participants having authored no SRs, 9 having authored 1–9 SRs, and 1 having authored more than 10 SRs (Table 2).

**Table 2:** Characteristics of participants who performed translations during the trial

Characteristics of trial participants	Participants (n=15*)

Levels of formal training (multiple selections possible)	

Bachelor’s and/or master’s in library science	10
Bachelor’s in non-library science	6
Master’s and/or doctorate (PhD) in nonlibrary science	4
Current position	
Librarian	13
Researcher or educator	2
Place of work	
University	14
Hospital	1
Years of systematic review (SR) searching experience	
10+ years	5
3–9 years	7
0–2 years	3
Number of SRs authored	
10+	1
5–9	4
1–4	5
0	5
Years of experience in searching the literature	
20+	6
10–19	4
0–9	5

* 15 of 16 participants who performed translations during the trial responded to the survey.

### Time taken to translate the search strings

**Figure 1:**
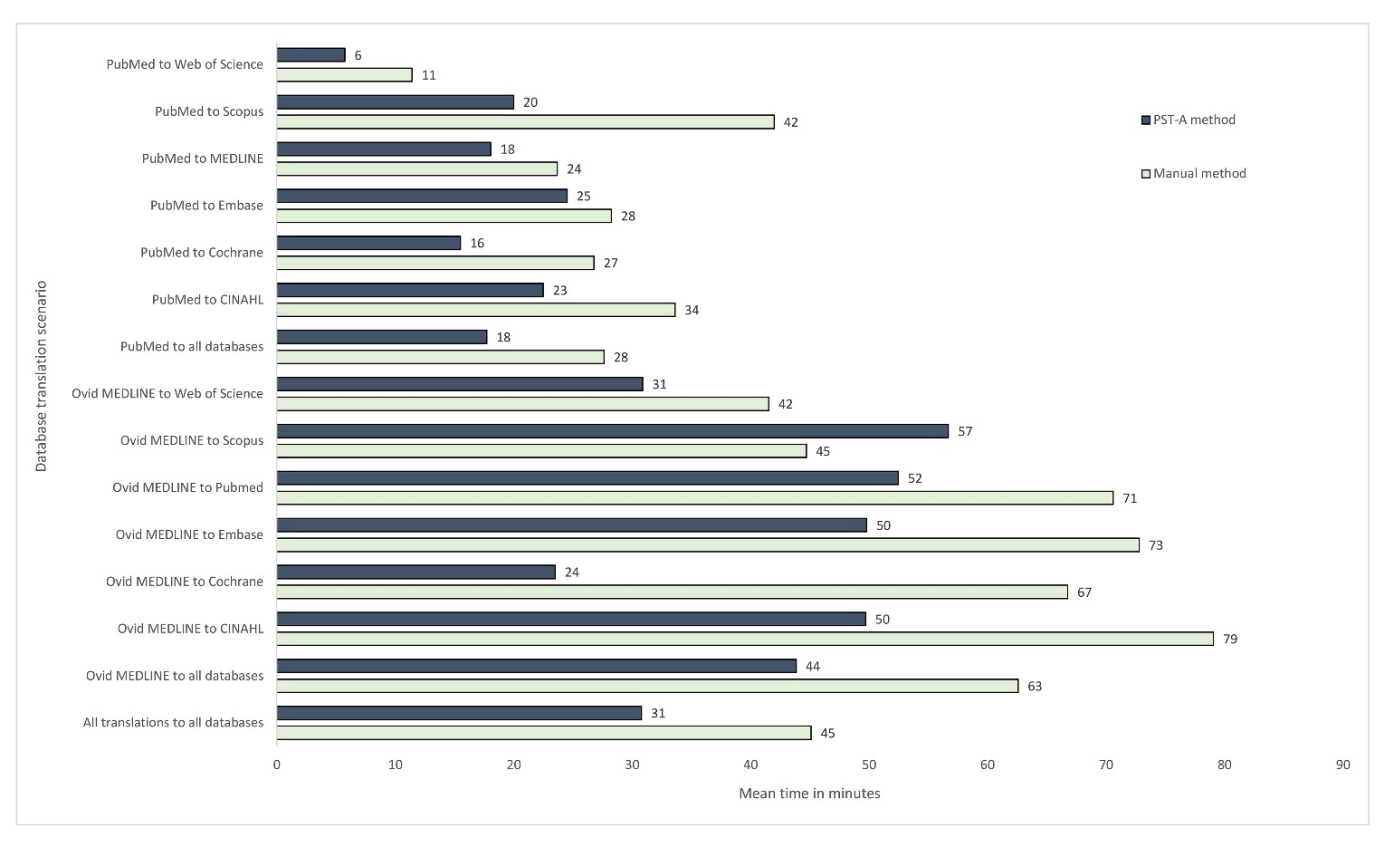
Mean time taken (minutes) to translate search strings by the Polyglot Search Translator–assisted (PST-A) and manual methods Abbreviation: PST-A=Polyglot Search Translator–assisted.

When all collected data were analysed, the PST-A method was faster than the manual method of translating search strings, with a mean time saving of 14 minutes (PST-A method, mean: 31, standard deviation (SD): 39; manual method, mean: 45, SD: 59) (Figure 1). The mean time saving for translating search strings originating from PubMed was 10 minutes and from Ovid MEDLINE was 19 minutes (supplemental Appendix F).

When analyzing data using the mixed linear model, there was insufficient evidence of an interaction between method and search string (*p*=0.37) or between method and specified translation databases (*p*=0.28); hence, these interaction terms were removed from the model. After adjustment for specified search string and translation databases, the PST-A method reduced the time taken to translate search strings by 32% (95% confidence interval [CI]: 22%–40%), compared with the manual method.

### Number of errors in the search translations

**Figure 2:**
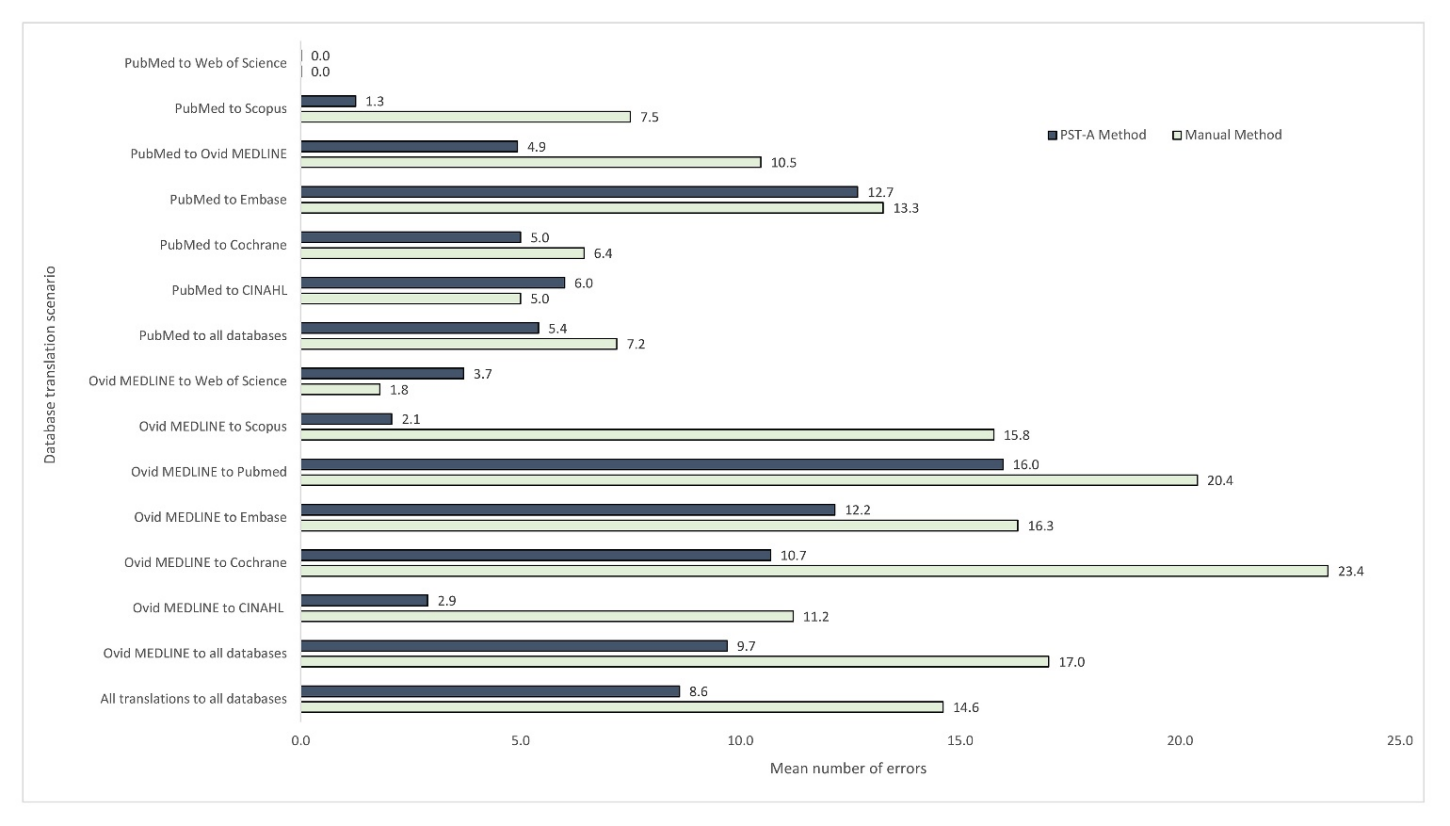
Mean number of errors by the PST-A and manual methods

When all collected data were analyzed, there was a mean of 8.6 errors (SD: 9) per translation by the PST-A method versus 14.6 errors (SD: 26) by the manual method (Figure 2). The mean number of errors affecting recall was 7 (SD: 7) with the PST-A method and 8 (SD: 19) with the manual method. The mean number of errors affecting precision was 1 (SD: 7) with the PST-A method and 6 (SD: 18) with the manual method (supplemental Appendix G). The PST-A method made fewer unique errors in 18 of the 32 error type categories, the manual method made fewer unique errors in 8 of the 32 error type categories, and the error rates were the same in 6 of the 32 error type categories (Table 3).

**Table 3:**
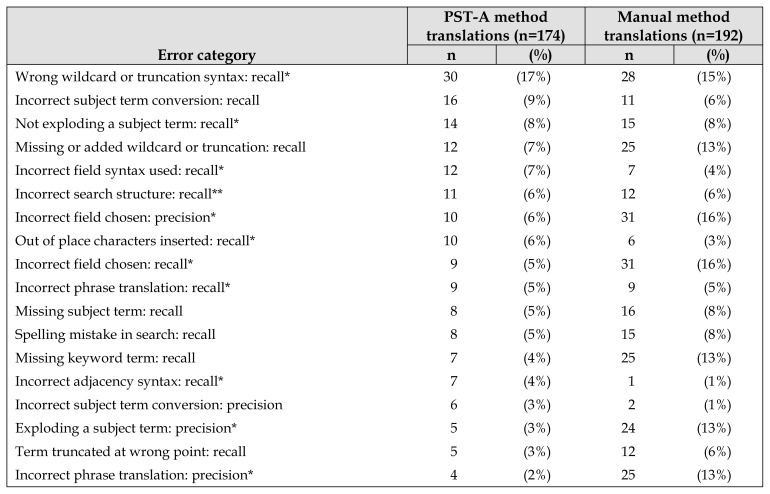
Number and percent of unique errors in search string translations

Error category	PST-A method translations (n=174)		Manual method translations (n=192)	

	n	(%)	n	(%)
Wrong wildcard or truncation syntax: recall*	30	(17%)	28	(15%)
Incorrect subject term conversion: recall	16	(9%)	11	(6%)
Not exploding a subject term: recall*	14	(8%)	15	(8%)
Missing or added wildcard or truncation: recall	12	(7%)	25	(13%)
Incorrect field syntax used: recall*	12	(7%)	7	(4%)
Incorrect search structure: recall^**^	11	(6%)	12	(6%)
Incorrect field chosen: precision*	10	(6%)	31	(16%)
Out of place characters inserted: recall*	10	(6%)	6	(3%)
Incorrect field chosen: recall*	9	(5%)	31	(16%)
Incorrect phrase translation: recall*	9	(5%)	9	(5%)
Missing subject term: recall	8	(5%)	16	(8%)
Spelling mistake in search: recall	8	(5%)	15	(8%)
Missing keyword term: recall	7	(4%)	25	(13%)
Incorrect adjacency syntax: recall*	7	(4%)	1	(1%)
Incorrect subject term conversion: precision	6	(3%)	2	(1%)
Exploding a subject term: precision*	5	(3%)	24	(13%)
Term truncated at wrong point: recall	5	(3%)	12	(6%)
Incorrect phrase translation: precision*	4	(2%)	25	(13%)
Incorrect adjacency used: precision	4	(2%)	9	(5%)
Other error type: recall	8	(4%)	9	(5%)
Other error type: precision	17	(8%)	18	(9%)
Total	206		329	

Abbreviation: PST-A=Polyglot Search Translator–assisted.

* Error identified and fixed in the PST after trial completion.

Mixed model analysis showed insufficient evidence of an interaction between method and translation databases specified for number of errors made (*p*=0.93). However, there was evidence of an interaction between translation method and search string (*p*=0.003). This means the effect of method on the number of errors made differed depending on which search string was being translated. In an exploratory analysis, the complexity of the search string was investigated as a possible explanatory variable.

Search strings were ranked from 1 to 20 for complexity, where 1=least complex and 20=most complex (supplemental Appendix E). This variable was centered at the mean and included in the model instead of search string. Adjusting for translation databases specified, on average, translations performed with the assistance of the PST reduced the number of errors by 45% (95% CI: 28%–58%); however, this effect diminished by 9% (95% CI: 4%–14%) for each increase in complexity by 1 rank score. This result means that the improvement in errors made using the PST-A method was greatest for less complex searches and least for more complex searches.

### Differences in the number of references retrieved

**Figure 3:**
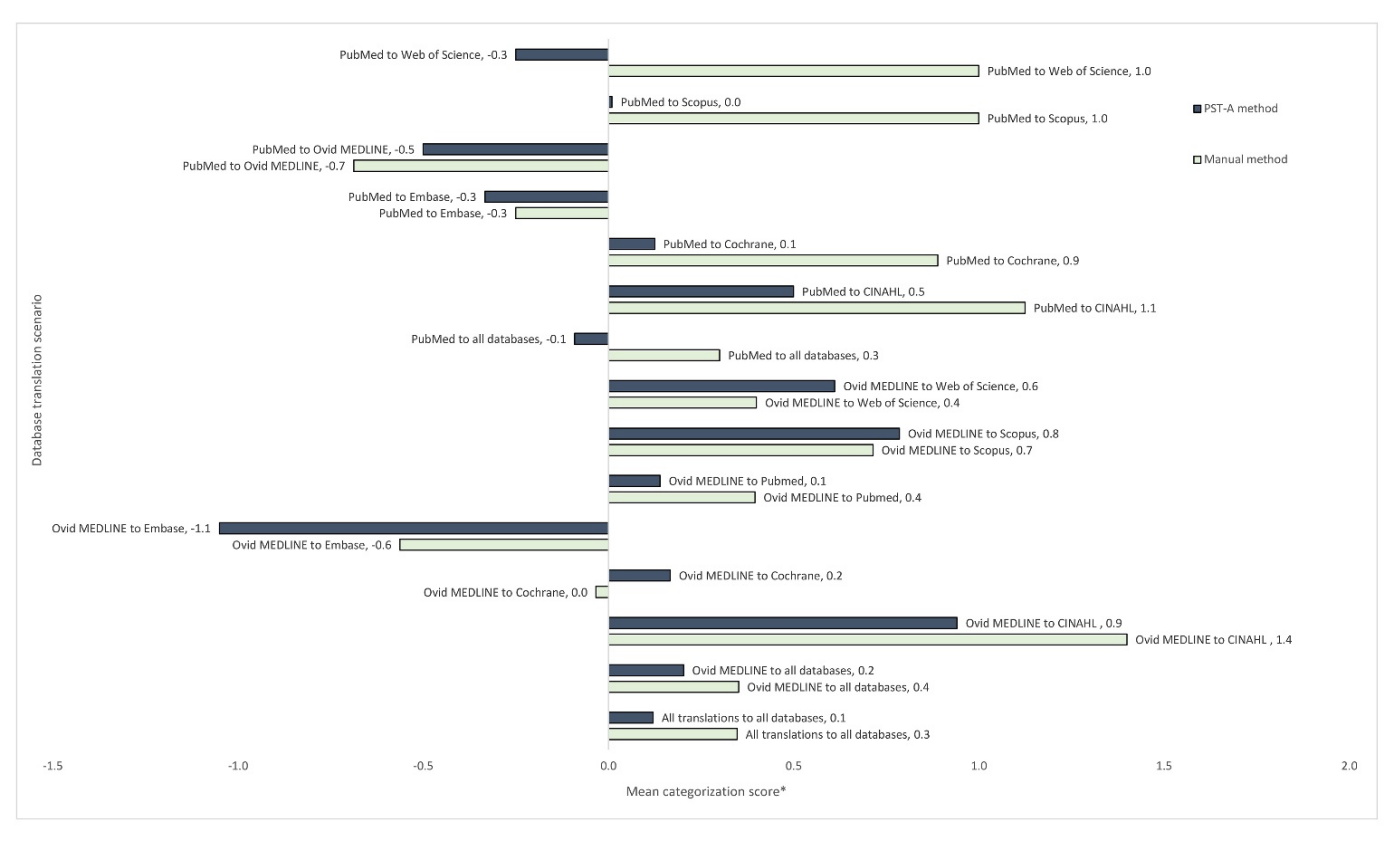
Mean categorization score for the number of references retrieved by translated search strings compared to the reference standard translation * The smaller the score (and the smaller the bar) the closer the number of references retrieved by the translated search to the number of references retrieved by the reference standard translation.

Large variation in the number of references retrieved made the results reported for this outcome inconclusive. However, we reported the results for completeness and transparency. When analyzing all collected data, the mean of the categorization score was 0.1 for the PST-A method and 0.3 for the manual method (Figure 3). The categorization score represented the deviation in the number of references retrieved by the translated search string from the expected number of references retrieved by the reference standard translation, with a score of −2 the largest negative deviation (likely to affect recall), +2 the largest positive deviation (many extra records to screen), and 0 no deviation. Median scores of numbers, with ranges, are provided in supplemental Appendix H.

The mixed model for closeness showed insufficient evidence of an interaction between method and search string (*p*=0.18) or between method and translation databases specified (*p*=0.84); hence, these interaction terms were removed from the model. After adjustment for search string and translation databases specified, PST improved closeness by 27% (95% CI: 16% worse–49% better), compared with the manual method (reference), but this improvement was not statistically significant (*p*=0.21).

## DISCUSSION

Across all translations, the PST-A method reduced the time taken to translate search strings by 14 minutes, which equated to a time saving of approximately 30%. The PST-A method also resulted in fewer errors, with a mean of 8.6 errors per translation versus 14.6 errors per translation by the manual method. Translation errors were still common, irrespective of the method used. As the complexity of the original search increased, the difference in the number of errors occurring between the translation methods reduced. In addition, the number of references retrieved by search strings translated by the PST-A method was closer to the number of references retrieved by the reference standard translation compared to the manual method, although wide variation in the data for this outcome made this finding an unreliable indicator of search translation quality.

Identifying studies to include in an SR involves searching multiple databases [1, 12], which can be time consuming and error prone [2, 3, 13–15]. The results of this study suggest the PST, when used as an aid to translate database search strings, can help with this problem. The time saving seen with the PST offers a substantial benefit for those performing searches for SRs. For an SR searching four databases [16], in which three database search string translations are required, use of the PST can save almost forty-five minutes of search time.

Across the databases, the PST-A method consistently saved time, with it being faster in 14 of the 15 search translation scenarios, the exception being translations from Ovid MEDLINE to Scopus. This might be due to Scopus not being as commonly used by clinical search specialists, meaning that any time-saving benefit of the PST could have been lost during the checking of the PST search for errors, something that is quicker and easier in a database with which a user is familiar. Time savings were more pronounced when translating searches from Ovid MEDLINE than when translating searches in PubMed format. This was most likely because Ovid MEDLINE searches tend to be more complex than PubMed searches. The most complex Ovid MEDLINE search had around 145 search terms, while the most complex PubMed search had 40 search terms. In other words, with more search terms to translate there is a greater time saving when automatically translating them.

Errors in search strings can have significant implications for recall (missing relevant studies) and precision (irrelevant studies need to be screened), both of which can substantially impact the findings of the SR and the resources required for its completion. This is an ongoing issue, with 73% of Cochrane reviews having at least 1 error in 2015 [13]. Errors in non-Cochrane reviews are harder to determine due to problems in the reporting of searches [17].

This study shows that the PST can reduce translation errors, as it made fewer errors in thirteen of the fifteen search translation scenarios; however, translation errors still occurred. The errors made by the PST in the trial (e.g., the use of an incorrect wildcard) have been fixed (highlighted by an * in Table 3), meaning the errors in future PST-A searches should be reduced. The last of the errors were fixed during the latest upgrade to the PST in October 2019. However, upgrades to the PST will not fix human-made errors, such as incorrect translations of MeSH to Emtree terms, so searchers need to be aware of this. Future ways to deal with these errors would be to make the PST alert searchers where manual translation is required, such as translating thesaurus terms, by highlighting them in the translated search string.

The PST appears to be particularly effective for reducing the number of precision errors. As SRs become more complex, the searches for them also become more complex, and these searches tend to return more references to screen. Therefore, precision errors can translate into substantially more irrelevant references to screen, meaning more work for authors, so any reduction in precision errors should translate into a time saving for SR authors.

In this study, the number of references retrieved by the translated search strings compared to the reference standard translation was originally considered to be an indicator of translation quality because it commonly is used to test searches [18–21]. However, variability in the data makes it difficult to draw useful conclusions, and the results for this outcome should be read cautiously. A main cause of this may be due to certain types of errors causing a far greater deviation from the numbers that should be found than others. For instance, if there is a missing bracket in a search string, this will normally cause a far greater impact than choosing the wrong field would.

Despite this unreliability, a couple of the findings are worth noting. For instance, when translating from Ovid MEDLINE to Embase, both methods produced translations that retrieved fewer studies than the number that was expected to be found; although this is a similar outcome, it was for different reasons. The PST-A method seems to have found less than it should have due to a single type of error: an incorrect wildcard translation that has now been corrected. The manual method seemed to find less than it should have due to many types of errors, such as focusing subject terms, applying database specific limits, and choosing the wrong fields. When translating from Ovid MEDLINE to CINAHL, both methods tended to find more than the number that was expected to be found. This was possibly because CINAHL searches tend to contain more brackets than searches in other databases, and a single wrong bracket can have a large impact on search results.

An important consideration when reviewing the results of this trial is that the participants were working in an experimental environment with search strings that they had not developed. In practice, participants would normally be translating searches that they designed themselves. Having designed the search, they would understand its logic and probably be more likely to spot errors in the translations. This means the error counts found in this study might be higher than what would occur in practice. Familiarity with the search strings would also impact the number of references retrieved due to the similarity between numbers of references retrieved being used as a guide to translation quality. How this familiarity with the search string might impact time saving is more difficult to determine, as it could either reduce or increase the benefit.

Other tools for translating searches exist [5–7] but have yet to be tested outside of the groups that developed them; therefore, their benefit is difficult to determine. The considerable effort put into developing these tools suggests that the search string translation step is one area where the quality and speed of SRs can be improved. Feedback from trial participants and users who were not involved in the trial is being used to improve the PST’s usability and reliability. Other larger initiatives, such as automatically generating single line search strings from numbered line searches and highlighting translations that require attention from the user, have been completed and will be included in version 3 of the PST, which was implemented in late 2019.

### Strengths and limitations

This study had several limitations. Most participants were from a library and information science background, making it difficult to generalize study applicability to other types of specialists. Loss of search string translations meant that the data were not completely balanced, and the search strings were translated out of the context of the original research question, meaning participants lacked the usual background knowledge that they would have on the topic and benchmarking numbers from the original search. In addition, the study was designed and run by the creators of the PST, but external recruitment of participants, random selection and allocation of search strings and methods, and blinding of the assessors was done to minimize bias as much as possible. Study strengths include the randomization of participants to the method of translation, recruitment of participants from outside of the group that developed the PST, random selection of published search strings, variability in the experience of the participants in conducting searches for SRs, and sufficient power of the study to reveal an effect of the intervention.

## CONCLUSION

The PST, when used as a tool to assist in the translation of search strings across multiple databases, can increase the speed of translation without an increase in errors. Errors in database search string translations remain a problem regardless of the assistance of the PST, and search specialists should be aware of this. These findings underpin the design philosophy of the PST: that the PST is *not* designed to replace the need for skilled people to translate search strings but rather to help skilled people translate search strings faster.

## DATA AVAILABILITY STATEMENT

Data are hosted on the Bond University repository: https://research.bond.edu.au/en/datasets/the-polyglot-search-translator-pst-evaluation-of-a-tool-for-impro.

## COMPETING INTERESTS

Author Justin Michael Clark has received awards, with prize money, to continue developing the Polyglot Search Translator from the Australian Library Information Association. All other authors declare that they have no other competing interests.

## FUNDING

This research was conducted with support from the Project Transform project at Cochrane Australia, funding for Project Transform was provided by Cochrane and the National Health and Medical Research Council of Australia (APP1114605). The contents of the published material are solely the responsibility of the administering institution, a participating institution, or individual authors and do not reflect the views of the National Health and Medical Research Council of Australia.

## SUPPLEMENTAL FILES

Appendix A Description of how the Polyglot Search Translator (PST) (intervention) works

Appendix B Results of searches conducted to find systematic review search strings used in the trial

Appendix C Systematic reviews whose search strategies were used in the trial

Appendix D Instructions provided to trial participants

Appendix E **Table S2** Search stings ranked by order of complexity (1=least complex, 20=most complex)

Appendix F **Table S3** Mean time, standard deviations, and mean differences in all times in minutes in search translations

Appendix G **Table S4** Totals, means, mean differences, and standard deviations in all errors in search translations; **Table S5** Totals, means, mean differences, and standard deviations in all recall errors in search translations; **Table S6** Totals, means, mean differences, and standard deviations in all precision errors in search translations; and **Table S7** Full list of number and percent of unique errors in search string translations

Appendix H **Table S8** Median percentage difference in the number of references retrieved relative to the number of references retrieved by the reference standard (with ranges)

## 

**Justin Michael Clark** (corresponding author), jclark@bond.edu.au, https://orcid.org/0000-0003-0133-1613, Institute for Evidence-Based Healthcare, Bond University, Robina, Queensland, Australia

**Sharon Sanders**, ssanders@bond.edu.au, Institute for Evidence-Based Healthcare, Bond University, Robina, Queensland, Australia

**Matthew Carter**, macarter@bond.edu.au, Institute for Evidence-Based Healthcare, Bond University, Robina, Queensland, Australia

**David Honeyman**, d_honeyman@hotmail.com, Bond University Library, Bond University, Robina, Queensland, Australia

**Gina Cleo**, gcleo@bond.edu.au, Institute for Evidence-Based Healthcare, Bond University, Robina, Queensland, Australia

**Yvonne Auld**, Yvonne.Auld@health.qld.gov.au, Gold Coast Health Library Service, Gold Coast University Hospital, Southport, Queensland, Australia

**Debbie Booth**, debbie.booth@newcastle.edu.au, University Library, University of Newcastle, Callaghan, New South Wales, Australia

**Patrick Condron**, p.condron@unimelb.edu.au, University Library, University of Melbourne, Melbourne, Victoria, Australia

**Christine Dalais**, c.dalais@library.uq.edu.au, University Library, University of Queensland, Brisbane, Queensland, Australia

**Sarah Bateup**, sbateup@bond.edu.au, Bond University Library, Bond University, Robina, Queensland, Australia

**Bronwyn Linthwaite**, blinthwa@bond.edu.au, Bond University Library, Bond University, Robina, Queensland, Australia

**Nikki May**, nikki.may@sa.gov.au, Sturt Library, Flinders University, Adelaide, South Australia, Australia

**Jo Munn**, Joanne.Munn@scu.edu.au, Centre for Teaching and Learning, Southern Cross University, Coffs Harbour, New South Wales, Australia

**Lindy Ramsay**, Lindy.Ramsay@acu.edu.au, University Library, Australian Catholic University, Banyo, Queensland, Australia

**Kirsty Rickett**, k.rickett@library.uq.edu.au, University Library, University of Queensland, Brisbane, Queensland, Australia

**Cameron Rutter**, c.rutter@qut.edu.au, University Library, Queensland University of Technology, Kelvin Grove, Queensland, Australia

**Angela Smith**, Angela.Smith@hnehealth.nsw.gov.au, Hunter New England Health Libraries, New South Wales (NSW) Health, Hunter Region, New South Wales, Australia

**Peter Sondergeld**, p.sondergeld@qut.edu.au, University Library, Queensland University of Technology, Kelvin Grove, Queensland, Australia

**Margie Wallin**, jam232@gmail.com, University Library, Southern Cross University, Coffs Harbour, New South Wales, Australia

**Mark Jones**, majones@bond.edu.au, Institute for Evidence-Based Healthcare, Bond University, Robina, Queensland, Australia

**Elaine Beller**, ebeller@bond.edu.au, Institute for Evidence-Based Healthcare, Bond University, Robina, Queensland, Australia
