# Supplementary material for: Improving the translation of search strategies using the Polyglot Search Translator: a randomized controlled trial
Source: J Med Libr Assoc. 2020 Apr 1;108(2):195–207. doi: 10.5195/jmla.2020.834 (PMC7069833; doi:10.5195/jmla.2020.834)

JMLA Journal of the Medical Library Association

SC
Supplemental content to
J Med Libr Assoc. Apr;108(2):dx.doi.org/10.5195/jmla.2020.834
www.jmla.mlanet.org
© Clark, Sanders, Carter, Honeyman, Cleo, Auld, Booth, Condron, Dalais, Bateup, Linthwaite, May, Munn, Ramsay, Rickett, Rutter, Smith, Sondergeld, Wallin, Jones, Beller 2020

Improving the translation of search strategies using the Polyglot Search Translator: a randomized controlled trial

Justin Michael Clark; Sharon Sanders; Matthew Carter; David Honeyman; Gina Cleo; Yvonne Auld; Debbie Booth; Patrick Condron; Christine Dalais; Sarah Bateup; Bronwyn Linthwaite; Nikki May; Jo Munn; Lindy Ramsay; Kirsty Rickett; Cameron Rutter; Angela Smith; Peter Sondergeld; Margie Wallin; Mark Jones; Elaine Beller

APPENDIX A

Description of how the Polyglot Search Translator (PST) (intervention) works

The Polyglot Search Translator (PST) is not designed to replace the need to understand how the database works: it is designed to help those with that knowledge achieve search translations faster. A key component that the PST does not do is to automatically map subject terms such as Medical Subject Headings (MeSH) terms across databases. Users still need to conduct this task themselves by looking up the appropriate thesaurus in each database.

To use the PST, copy and paste either a PubMed or Ovid MEDLINE into the "Your query" box, then click on the arrow next to database name to reveal the translated search string. Copy this search string into the search box of the appropriate database. If the search does not run as expected, you can paste it into a Word document and make any required edits (for instance, if you have not changed over the MeSH subject term to the subject term of the new database). More details on how the PST works can be found from the PST help guide, available at crebp-sra.com/#/help/polyglot.

The PST is a free tool that can be accessed from crebp-sra.com/#/polyglot and is available as MIT-licensed open source software at https://github.com/CREBP/sra-polyglot.

For example, the PST replaces the Ovid MEDLINE adjacency operator "ADJ" with "NEAR" when translating the search string to the Cochrane Library, and the truncation symbol "?" with "*" when translating search strings from Ovid to PubMed (Figure S1 and Figure S2). Thesaurus terms need to be manually translated by the searcher. Search strings can be translated from either, PubMed or Ovid MEDLINE, into the PubMed, Ovid MEDLINE/Ovid Embase/Ovid PsycINFO, Embase.com, Cochrane Library, Web of Science, Scopus, or CINAHL databases.

Figure S1 Screenshot of the Polyglot Search Translator (PST) translating a PubMed search to the Cochrane Library

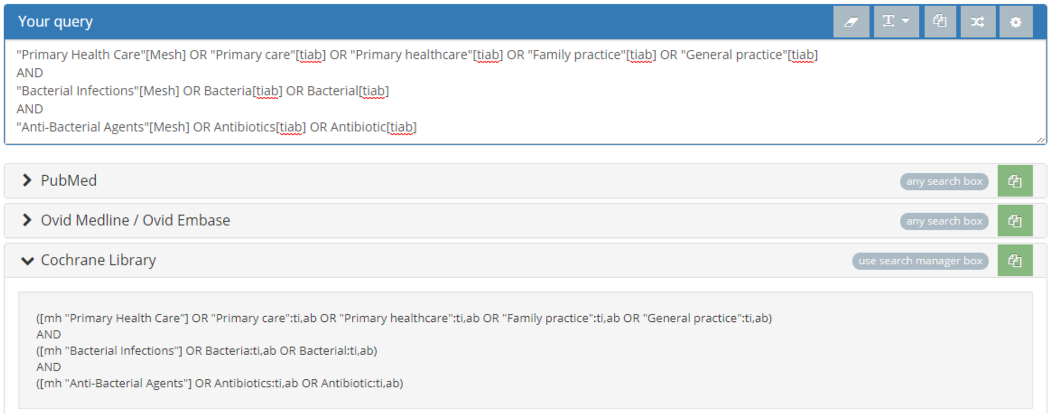

JMLA Journal of the Medical Library Association

Supplemental content to
J Med Libr Assoc. Apr;108(2):dx.doi.org/10.5195/jmla.2020.834
www.jmla.mlanet.org
© Clark, Sanders, Carter, Honeyman, Cleo, Auld, Booth, Condron, Dalais, Bateup, Linthwaite, May, Munn, Ramsay, Rickett, Rutter, Smith, Sondergeld, Wallin, Jones, Beller 2020

Figure S2 Screenshot of the PST translating an Ovid MEDLINE search to Embase.com

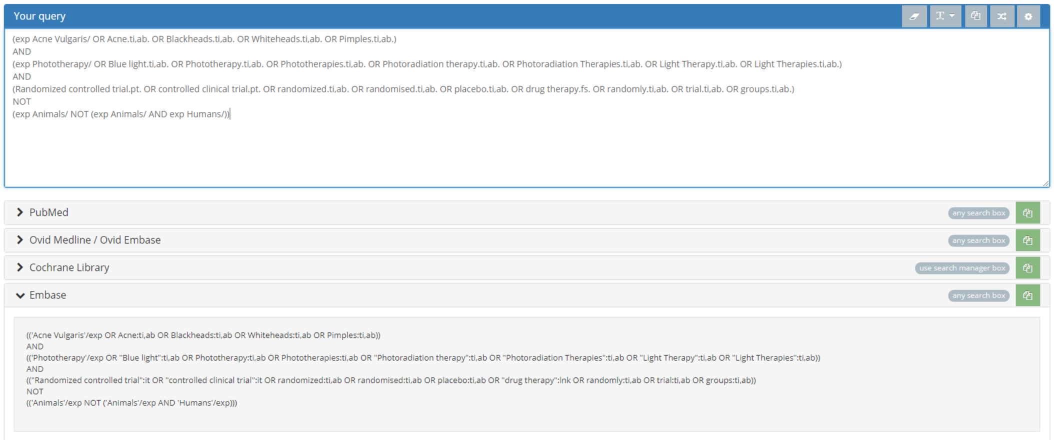

Supplement: Appendix A [file jmla-108-195-s001.pdf]
