# Supplementary material for: Improving the translation of search strategies using the Polyglot Search Translator: a randomized controlled trial
Source: J Med Libr Assoc. 2020 Apr 1;108(2):195–207. doi: 10.5195/jmla.2020.834 (PMC7069833; doi:10.5195/jmla.2020.834)
Supplement: Appendix B [file jmla-108-195-s002.pdf]

JMLA Journal of the Medical Library Association

Supplemental content to
J Med Libr Assoc. Apr;108(2):dx.doi.org/10.5195/jmla.2020.834
www.jmla.mlanet.org
© Clark, Sanders, Carter, Honeyman, Cleo, Auld, Booth, Condron, Dalais, Bateup, Linthwaite, May, Munn, Ramsay, Rickett, Rutter, Smith, Sondergeld, Wallin, Jones, Beller 2020

Improving the translation of search strategies using the Polyglot Search Translator: a randomized controlled trial

Justin Michael Clark; Sharon Sanders; Matthew Carter; David Honeyman; Gina Cleo; Yvonne Auld; Debbie Booth; Patrick Condron; Christine Dalais; Sarah Bateup; Bronwyn Linthwaite; Nikki May; Jo Munn; Lindy Ramsay; Kirsty Rickett; Cameron Rutter; Angela Smith; Peter Sondergeld; Margie Wallin; Mark Jones; Elaine Beller

APPENDIX B

Table S1 Results of searches conducted to find systematic review search strings used in the trial

Search strategy used to identify the reviews	Number of reviews screened	Number of reviews used in the trial	
Cochrane intervention reviews			
"Cochrane Database Syst Rev"[Journal] (PubMed, limited to last 5 years)	17	5	(1–5)
Drug intervention review			
"systematic review"[Title] AND intervention[Title/Abstract] NOT "Cochrane Database Syst Rev"[Journal] (PubMed, limited to last 5 years)	46	2	(6, 7)
Non-drug intervention reviews			
"systematic review"[Title] AND intervention[Title/Abstract] NOT "Cochrane Database Syst Rev"[Journal] (PubMed, limited to last 5 years)	59	3	(8–10)
Diagnostic reviews			
"systematic review"[Title] AND diagnostic[Title/Abstract] NOT "Cochrane Database Syst Rev"[Journal] (PubMed, limited to last 5 years)	29	3	(11–13)
Diagnostic reviews			
"systematic review"[Title] AND prevalence[Title/Abstract] NOT "Cochrane Database Syst Rev"[Journal] (PubMed, limited to last 5 years)	21	2	(14, 15)

JMLA Journal of the Medical Library Association

Supplemental content to
J Med Libr Assoc. Apr;108(2):dx.doi.org/10.5195/jmla.2020.834
www.jmla.mlanet.org
© Clark, Sanders, Carter, Honeyman, Cleo, Auld, Booth, Condron, Dalais, Bateup, Linthwaite, May, Munn, Ramsay, Rickett, Rutter, Smith, Sondergeld, Wallin, Jones, Beller 2020

Search strategy used to identify the reviews	Number of reviews screened	Number of reviews used in the trial	
Prognostic reviews			
"systematic review"[Title] AND prognosis[Title/Abstract] NOT "Cochrane Database Syst Rev"[Journal] PubMed, (limited to last 5 years)	9	2	(16, 17)
Health technology assessments			
(Health Technology Assessment Database (HTA), https://www.crd.york.ac.uk/CRDWeb/, (*), limited to 2012 to 2017, and published HTAs)	20	3	(18–20)
