# Supplementary material for: Improving the translation of search strategies using the Polyglot Search Translator: a randomized controlled trial
Source: J Med Libr Assoc. 2020 Apr 1;108(2):195–207. doi: 10.5195/jmla.2020.834 (PMC7069833; doi:10.5195/jmla.2020.834)
Supplement: Appendix C [file jmla-108-195-s003.pdf]

JMLA Journal of the Medical Library Association

SC

Supplemental content to
J Med Libr Assoc. Apr;108(2):dx.doi.org/10.5195/jmla.2020.834
www.jmla.mlanet.org
© Clark, Sanders, Carter, Honeyman, Cleo, Auld, Booth, Condron, Dalais, Bateup, Linthwaite, May, Munn, Ramsay, Rickett, Rutter, Smith, Sondergeld, Wallin, Jones, Beller 2020

Improving the translation of search strategies using the Polyglot Search Translator: a randomized controlled trial

Justin Michael Clark; Sharon Sanders; Matthew Carter; David Honeyman; Gina Cleo; Yvonne Auld; Debbie Booth; Patrick Condron; Christine Dalais; Sarah Bateup; Bronwyn Linthwaite; Nikki May; Jo Munn; Lindy Ramsay; Kirsty Rickett; Cameron Rutter; Angela Smith; Peter Sondergeld; Margie Wallin; Mark Jones; Elaine Beller

APPENDIX C

Systematic reviews whose search strategies were used in the trial

1. Alldred SK, Takwoingi Y, Guo B Pennant M, Deeks JJ, Neilson JP, Alfirevic Z. First and second trimester serum tests with and without first trimester ultrasound tests for Down's syndrome screening. Cochrane Database Syst Rev. 2017 Mar 15;3:CD012599.
2. Attridge M, Creamer J, Ramsden M, Cannings-John R, Hawthorne K. Culturally appropriate health education for people in ethnic minority groups with type 2 diabetes mellitus. Cochrane Database Syst Rev. 2014 Sep 4;(9):CD006424.
3. Crawford F, Andras A, Welch K, Sheares K, Keeling D, Chappell FM. D-dimer test for excluding the diagnosis of pulmonary embolism. Cochrane Database Syst Rev. 2016 Aug 5;(8):CD01086.
4. Palmer SC, Palmer AR, Craig JC, Johnson DW, Stroumza P, Frantzen L, Leal M, Hoischen S, Hegbrant J, Strippoli GF. Home versus in-centre haemodialysis for end-stage kidney disease. Cochrane Database Syst Rev. 2014 Nov 20;(11):CD009535.
5. Vaughan J, Nagendran M, Cooper J, Davidson BR, Gurusamy KS. Anaesthetic regimens for day-procedure laparoscopic cholecystectomy. Cochrane Database Syst Rev. 2014 Jan 24;(1):CD009784.
6. Nagler EV, Webster AC, Vanholder R, Zoccali C. Antidepressants for depression in stage 3–5 chronic kidney disease: a systematic review of pharmacokinetics, efficacy and safety with recommendations by European Renal Best Practice (ERBP). Nephrol Dial Transplant. 2012 Oct;27(10):3736–45.
7. Trinh KV, Kim J, Ritsma A. Effect of pseudoephedrine in sport: a systematic review. BMJ Open Sport Exercise Med. 2015 Dec 21;1(1):e000066.
8. Fredericks S, Yau T. Clinical effectiveness of individual patient education in heart surgery patients: a systematic review and meta-analysis. Int J Nurs Studies. 2017 Jan;65:44–53.
9. Pearce EE, Evenson KR, Downs DS, Steckler A. Strategies to promote physical activity during pregnancy: a systematic review of intervention evidence. Am J Lifestyle Med. 2013 Jan;7(1).
10. van Gils A, Schoevers RA, Bonvanie IJ, Gelauff JM, Roest AM, Rosmalen JG. Self-help for medically unexplained symptoms: a systematic review and meta-analysis. Psychosom Med. 2016 Jul–Aug;78(6):728–39.
11. Cadieux G, Campbell J, Dendukuri N. Systematic review of the accuracy of antibody tests used to screen asymptomatic adults for hepatitis C infection. CMAJ Open. 2016 Dec;4(4):E737–E745.
12. Calvo I, Conway A, Henriques F, Walshe M. Diagnostic accuracy of the clinical feeding evaluation in detecting aspiration in children: a systematic review. Dev Med Child Neurol. 2016 Jun;58(6):541–53.

Supplemental content to
J Med Libr Assoc. Apr;108(2):dx.doi.org/10.5195/jmla.2020.834
www.jmla.mlanet.org
© Clark, Sanders, Carter, Honeyman, Cleo, Auld, Booth, Condron, Dalais, Bateup, Linthwaite, May, Munn, Ramsay, Rickett, Rutter, Smith, Sondergeld, Wallin, Jones, Beller 2020

13. Vaidya A, Joore MA, ten Cate-Hoek AJ, Kleinegris MC, ten Cate H, Severens JL. A systematic review of model-based economic evaluations of diagnostic and therapeutic strategies for lower extremity artery disease. Thrombosis Haemostasis. 2014 Jan;111(1):19–28.

14. Rossi C, Shrier I, Marshall L, Cnossen S, Schwartzman K, Klein MB, Schwarzer G, Greenaway C. Seroprevalence of chronic hepatitis B virus infection and prior immunity in immigrants and refugees: a systematic review and meta-analysis. PLoS One. 2012;7(9):e44611.

15. Sapkota S, Brien JA, Greenfield J, Aslani P. A systematic review of interventions addressing adherence to anti-diabetic medications in patients with type 2 diabetes—impact on adherence. PLoS One. 2015 Feb 24;10(2):e0118296.

16. Eggerding V, Meuffels DE, Bierma-Zeinstra SM, Verhaar JA, Reijman M. Factors related to the need for surgical reconstruction after anterior cruciate ligament rupture: a systematic review of the literature. J Orthop Sports Phys Ther. 2015 Jan;45(1):37–44.

17. Lahart IM, Metsios GS, Nevill AM, Carmichael AR. Physical activity, risk of death and recurrence in breast cancer survivors: a systematic review and meta-analysis of epidemiological studies. Acta Oncol. 2015 May;54(5):635–54.

18. Althaus CL, Turner KM, Mercer CH, Auguste P, Roberts TE, Bell G, Herzog SA, Cassell JA, Edmunds WJ, White PJ, Ward H, Low N. Effectiveness and cost-effectiveness of traditional and new partner notification technologies for curable sexually transmitted infections: observational study, systematic reviews and mathematical modelling. Health Technol Assess. 2014 Jan;18(2):1–100, vii–viii.

19. Butler M, McCreedy E, Schwer N, Burgess D, Call K, Przedworski J, Rosser S, Larson S, Allen M, Fu S, Kane RL. AHRQ comparative effectiveness reviews. improving cultural competence to reduce health disparities. Rockville (MD): Agency for Healthcare Research and Quality (US); 2016.

20. Wardlaw J, Brazzelli M, Miranda H, Chappell F, McNamee P, Scotland G, Quayyum Z, Martin D, Shuler K, Sandercock P, Dennis M. An assessment of the cost-effectiveness of magnetic resonance, including diffusion-weighted imaging, in patients with transient ischaemic attack and minor stroke: a systematic review, meta-analysis and economic evaluation. Health Technol Assess. 2014 Apr;18(27):1–368, v–vi. Erratum: Wardlaw J, Brazzelli M, Miranda H, Chappell F, McNamee P, Scotland G, Quayyum Z, Martin D, Shuler K, Sandercock P, Dennis M. Erratum: an assessment of the cost-effectiveness of magnetic resonance, including diffusion-weighted imaging, in patients with transient ischaemic attack and minor stroke: a systematic review, meta-analysis and economic evaluation. Health Technol Assess. 2015 Sep;18(27):369–70.
