# Supplementary material for: Improving the translation of search strategies using the Polyglot Search Translator: a randomized controlled trial
Source: J Med Libr Assoc. 2020 Apr 1;108(2):195–207. doi: 10.5195/jmla.2020.834 (PMC7069833; doi:10.5195/jmla.2020.834)
Supplement: Appendix D [file jmla-108-195-s004.pdf]

JMLA Journal of the Medical Library Association

SC

Supplemental content to
J Med Libr Assoc. Apr;108(2):dx.doi.org/10.5195/jmla.2020.834
www.jmla.mlanet.org
© Clark, Sanders, Carter, Honeyman, Cleo, Auld, Booth, Condron, Dalais, Bateup, Linthwaite, May, Munn, Ramsay, Rickett, Rutter, Smith, Sondergeld, Wallin, Jones, Beller 2020

Improving the translation of search strategies using the Polyglot Search Translator: a randomized controlled trial

Justin Michael Clark; Sharon Sanders; Matthew Carter; David Honeyman; Gina Cleo; Yvonne Auld; Debbie Booth; Patrick Condron; Christine Dalais; Sarah Bateup; Bronwyn Linthwaite; Nikki May; Jo Munn; Lindy Ramsay; Kirsty Rickett; Cameron Rutter; Angela Smith; Peter Sondergeld; Margie Wallin; Mark Jones; Elaine Beller

APPENDIX D

Instructions provided to trial participants

You will receive ten searches to translate. These are to be translated in the order received using the method specified for each (manual or using the Polyglot Search Translator [PST]).

Each search will be either a PubMed search or an Ovid MEDLINE search, and you will have been allocated three databases to translate the search into.

For each translation:

Timed elements:

- Record the amount of time (either manually or using the PST) it takes you to translate and run the search in each database.
- Also record the amount of time spent:
  - Reading help files to understand how the databases work
  - Watching online videos or doing online tutorials
  - Meetings with support personnel or gaining help from experts
- If you are interrupted for more than five minutes during this process, please pause your timer.

Non-timed elements:

- Record the number of search results your translated search finds in each database.
- Save your translated search in each database.
- Record your actual translated search strategy for each database; this can be in the Excel spreadsheet or a separate Word document.

Please send your timing results spreadsheet, ten compressed EndNote libraries, and spreadsheet or Word document with translated search strategies to the research team.

Notes:

Limitations of databases

Your aim is to translate each search to run as close to the original on each database. The different databases have different capabilities in terms of search syntax, subject headings, etc., so you may not be able to replicate all the features of the original search as you translate. Use your judgment and the available help to decide how to translate each search to run as effectively as you can.

Improving the PST and the PST help guides

As well as being a trial, this is also a project in quality improvement for the PST and accompanying help guide. So please record any issues you had or thoughts on how to improve it or the help guide. Importantly, do NOT record the time spent doing this.

JMLA Journal of the Medical Library Association

SC

Supplemental content to
J Med Libr Assoc. Apr;108(2):dx.doi.org/10.5195/jmla.2020.834
www.jmla.mlanet.org
© Clark, Sanders, Carter, Honeyman, Cleo, Auld, Booth, Condron, Dalais, Bateup, Linthwaite, May, Munn, Ramsay, Rickett, Rutter, Smith, Sondergeld, Wallin, Jones, Beller 2020

Translating using the PST

The PST is intended to be a tool to assist searchers but does not replace the need for the user to have knowledge of the searching process. You may need to modify or add to the translations produced by the PST using your own knowledge; ensure you record the time spent doing this.

Seeking help

You can seek help from any available sources (e.g., the database help guides, Google, or contacting someone). The only limitation placed on this is you CANNOT seek help from someone else involved in the trial. The trial coordinator is available to help at any time with either searching-related questions or trial methods questions.
