# Supplementary material for: Improving the translation of search strategies using the Polyglot Search Translator: a randomized controlled trial
Source: J Med Libr Assoc. 2020 Apr 1;108(2):195–207. doi: 10.5195/jmla.2020.834 (PMC7069833; doi:10.5195/jmla.2020.834)
Supplement: Appendix E [file jmla-108-195-s005.pdf]

JMLA Journal of the Medical Library Association

Supplemental content to
J Med Libr Assoc. Apr;108(2):dx.doi.org/10.5195/jmla.2020.834
www.jmla.mlanet.org
© Clark, Sanders, Carter, Honeyman, Cleo, Auld, Booth, Condron, Dalais, Bateup, Linthwaite, May, Munn, Ramsay, Rickett, Rutter, Smith, Sondergeld, Wallin, Jones, Beller 2020

Improving the translation of search strategies using the Polyglot Search Translator: a randomized controlled trial

Justin Michael Clark; Sharon Sanders; Matthew Carter; David Honeyman; Gina Cleo; Yvonne Auld; Debbie Booth; Patrick Condron; Christine Dalais; Sarah Bateup; Bronwyn Linthwaite; Nikki May; Jo Munn; Lindy Ramsay; Kirsty Rickett; Cameron Rutter; Angela Smith; Peter Sondergeld; Margie Wallin; Mark Jones; Elaine Beller

APPENDIX E

Table S2 Search stings ranked by order of complexity (1=least complex, 20=most complex)

Search no.*	Search string	Complexity
8	Attridge 2014 [2]	20
10	Butler 2016 [19]	19
2	Crawford 2016 [3]	18
4	Fredericks 2017 [8]	17
15	Alldred 2017 [1]	16
7	Nagler 2012 [6]	15
13	Cadieux 2016 [11]	14
11	Wardlaw 2014 [20]	13
17	Calvo 2016 [12]	12
19	Vaidya 2014 [13]	11
16	Eggerding 2015 [16]	10
6	Sapkota 2015 [15]	9
14	Trinh 2015 [7]	8
5	Palmer 2014 [4]	7
3	Lahart 2015 [17]	6
1	van Gils 2016 [10]	5
20	Althaus 2014 [18]	4
12	Vaughan 2014 [5]	3
18	Rossi 2012 [14]	2
9	Pearce 2013 [9]	1

* Reference numbers (e.g., [2]) taken from supplemental Appendix C.
