# Supplementary material for: Improving the translation of search strategies using the Polyglot Search Translator: a randomized controlled trial
Source: J Med Libr Assoc. 2020 Apr 1;108(2):195–207. doi: 10.5195/jmla.2020.834 (PMC7069833; doi:10.5195/jmla.2020.834)
Supplement: Appendix F [file jmla-108-195-s006.pdf]

JMLA Journal of the Medical Library Association

Supplemental content to
J Med Libr Assoc. Apr;108(2):dx.doi.org/10.5195/jmla.2020.834
www.jmla.mlanet.org
© Clark, Sanders, Carter, Honeyman, Cleo, Auld, Booth, Condron, Dalais, Bateup, Linthwaite, May, Munn, Ramsay, Rickett, Rutter, Smith, Sondergeld, Wallin, Jones, Beller 2020

Improving the translation of search strategies using the Polyglot Search Translator: a randomized controlled trial

Justin Michael Clark; Sharon Sanders; Matthew Carter; David Honeyman; Gina Cleo; Yvonne Auld; Debbie Booth; Patrick Condron; Christine Dalais; Sarah Bateup; Bronwyn Linthwaite; Nikki May; Jo Munn; Lindy Ramsay; Kirsty Rickett; Cameron Rutter; Angela Smith; Peter Sondergeld; Margie Wallin; Mark Jones; Elaine Beller

APPENDIX F

Table S3 Mean time, standard deviations, and mean differences in all times in minutes in search translations

Translation scenario	PST-A method translation time (minutes)		Manual method translation time (minutes)		Mean difference
	Mean	SD	Mean	SD	
PubMed to Web of Science	6	5	11	8	–6
PubMed to Scopus	20	20	42	47	–22
PubMed to MEDLINE	18	14	24	13	–6
PubMed to Embase	25	15	28	23	–4
PubMed to Cochrane	16	12	27	14	–11
PubMed to CINAHL	23	16	34	28	–11
PubMed to all databases	18	14	28	21	–10
Ovid MEDLINE to Web of Science	31	27	42	38	–11
Ovid MEDLINE to Scopus	57	55	45	31	12
Ovid MEDLINE to PubMed	52	41	71	71	–18
Ovid MEDLINE to Embase	50	57	73	67	–23
Ovid MEDLINE to Cochrane	24	17	67	60	–43
Ovid MEDLINE to CINAHL	50	44	79	99	–29
Ovid MEDLINE to all databases	44	43	63	67	–19
All databases	31	39	45	59	–14

Abbreviation: PST-A=Polyglot Search Translator–assisted; SD=Standard deviation.
