# Supplementary material for: Improving the translation of search strategies using the Polyglot Search Translator: a randomized controlled trial
Source: J Med Libr Assoc. 2020 Apr 1;108(2):195–207. doi: 10.5195/jmla.2020.834 (PMC7069833; doi:10.5195/jmla.2020.834)
Supplement: Appendix G [file jmla-108-195-s007.pdf]

JMLA Journal of the Medical Library Association

Supplemental content to
J Med Libr Assoc. Apr;108(2):dx.doi.org/10.5195/jmla.2020.834
www.jmla.mlanet.org
© Clark, Sanders, Carter, Honeyman, Cleo, Auld, Booth, Condron, Dalais, Bateup, Linthwaite, May, Munn, Ramsay, Rickett, Rutter, Smith, Sondergeld, Wallin, Jones, Beller 2020

Improving the translation of search strategies using the Polyglot Search Translator: a randomized controlled trial

Justin Michael Clark; Sharon Sanders; Matthew Carter; David Honeyman; Gina Cleo; Yvonne Auld; Debbie Booth; Patrick Condron; Christine Dalais; Sarah Bateup; Bronwyn Linthwaite; Nikki May; Jo Munn; Lindy Ramsay; Kirsty Rickett; Cameron Rutter; Angela Smith; Peter Sondergeld; Margie Wallin; Mark Jones; Elaine Beller

APPENDIX G

Table S4 Totals, means, mean differences, and standard deviations in all errors in search translations

Translation scenario	No. of PST-A translated searches	No. of manual translated searches	No. of PST-A errors	No. of manual errors	PST-A errors Mean	SD	Manual errors Mean	SD	Mean difference
PubMed to Web of Science	4	7	0	0	0	0	0	0	0
PubMed to Scopus	4	4	5	30	1	1	8	12	–7
PubMed to Ovid MEDLINE	14	15	69	157	5	11	10	13	–5
PubMed to Embase	6	4	76	53	13	18	13	15	0
PubMed to Cochrane	8	9	40	58	5	13	6	12	–1
PubMed to CINAHL	8	8	48	40	6	9	5	8	1
PubMed to all databases	44	47	238	338	5	11	7	11	2
Ovid MEDLINE to Web of Science	17	15	63	27	4	11	2	3	2
Ovid MEDLINE to Scopus	14	21	29	331	2	3	16	27	–14
Ovid MEDLINE to PubMed	42	48	671	979	16	24	20	32	–4
Ovid MEDLINE to Embase	20	16	243	261	12	20	16	26	–4
Ovid MEDLINE to Cochrane	19	30	203	701	11	18	23	35	–12

JMLA Journal of the Medical Library Association

Supplemental content to
J Med Libr Assoc. Apr;108(2):dx.doi.org/10.5195/jmla.2020.834
www.jmla.mlanet.org
© Clark, Sanders, Carter, Honeyman, Cleo, Auld, Booth, Condron, Dalais, Bateup, Linthwaite, May, Munn, Ramsay, Rickett, Rutter, Smith, Sondergeld, Wallin, Jones, Beller 2020

Translation scenario	No. of PST-A translated searches	No. of manual translated searches	No. of PST-A errors	No. of manual errors	PST-A errors Mean	SD	Manual errors Mean	SD	Mean difference
Ovid MEDLINE to CINAHL	18	15	52	168	3	4	11	18	–8
Ovid MEDLINE to all databases	130	145	1,261	2,467	10	18	17	29	–7
All translations to all databases	174	192	1,499	2,805	9	17	15	26	–6

Abbreviation: PST-A=Polyglot Search Translator–assisted; SD=Standard deviation.

JMLA Journal of the Medical Library Association

Supplemental content to
J Med Libr Assoc. Apr;108(2):dx.doi.org/10.5195/jmla.2020.834
www.jmla.mlanet.org
© Clark, Sanders, Carter, Honeyman, Cleo, Auld, Booth, Condron, Dalais, Bateup, Linthwaite, May, Munn, Ramsay, Rickett, Rutter, Smith, Sondergeld, Wallin, Jones, Beller 2020

Table S5 Totals, means, mean differences, and standard deviation in all recall errors in search translations

Translation scenario	No. of PST-A translated searches	No. of manual translated searches	No. of PST-A recall errors	No. of manual recall errors	PST-A recall errors Mean	SD	Manual recall errors Mean	SD	Mean difference
PubMed to Web of Science	4	7	0	0	0	0	0	0	0
PubMed to Scopus	4	4	5	0	1	1	0	0	1
PubMed to Ovid MEDLINE	14	15	67	156	5	11	10	13	–5
PubMed to Embase	6	4	76	52	13	18	13	16	0
PubMed to Cochrane	8	9	40	57	5	13	6	12	–1
PubMed to CINAHL	8	8	39	16	5	9	2	3	3
PubMed to all databases	44	47	227	281	5	11	6	11	–1
Ovid MEDLINE to Web of Science	17	15	55	9	3	11	1	1	2
Ovid MEDLINE to Scopus	14	21	25	72	2	3	3	6	–1
Ovid MEDLINE to PubMed	42	48	536	718	13	24	15	29	–2
Ovid MEDLINE to Embase	20	16	222	160	11	20	10	18	1
Ovid MEDLINE to Cochrane	19	30	160	242	8	18	8	19	0
Ovid MEDLINE to CINAHL	18	15	48	116	3	4	8	16	–5

JMLA Journal of the Medical Library Association

Supplemental content to
J Med Libr Assoc. Apr;108(2):dx.doi.org/10.5195/jmla.2020.834
www.jmla.mlanet.org
© Clark, Sanders, Carter, Honeyman, Cleo, Auld, Booth, Condron, Dalais, Bateup, Linthwaite, May, Munn, Ramsay, Rickett, Rutter, Smith, Sondergeld, Wallin, Jones, Beller 2020

Translation scenario	No. of PST-A translated searches	No. of manual translated searches	No. of PST-A recall errors	No. of manual recall errors	PST-A recall errors Mean	SD	Manual recall errors Mean	SD	Mean difference
Ovid MEDLINE to all databases	130	145	1,046	1,317	8	18	9	21	–1
All translations to all databases	174	192	1,273	1,598	7	17	8	19	–1

Abbreviation: PST-A=Polyglot Search Translator–assisted; SD=Standard deviation.

JMLA Journal of the Medical Library Association

Supplemental content to
J Med Libr Assoc. Apr;108(2):dx.doi.org/10.5195/jmla.2020.834
www.jmla.mlanet.org
© Clark, Sanders, Carter, Honeyman, Cleo, Auld, Booth, Condron, Dalais, Bateup, Linthwaite, May, Munn, Ramsay, Rickett, Rutter, Smith, Sondergeld, Wallin, Jones, Beller 2020

Table S6 Totals, means, mean differences, and standard deviation in all precision errors in search translations

Translation scenario	No. of PST-A translated searches	No. of manual translated searches	No. of PST-A recall errors	No. of manual recall errors	PST-A recall errors Mean	SD	Manual recall errors Mean	SD	Mean difference
PubMed to Web of Science	4	7	0	0	0	0	0	0	0
PubMed to Scopus	4	4	0	30	0	0	8	12	–8
PubMed to Ovid MEDLINE	14	15	2	1	0	0	0	0	0
PubMed to Embase	6	4	0	1	0	0	0	1	0
PubMed to Cochrane	8	9	0	1	0	0	0	0	0
PubMed to CINAHL	8	8	9	24	1	3	3	8	–2
PubMed to all databases	44	47	11	57	0	1	1	5	–1
Ovid MEDLINE to Web of Science	17	15	8	18	0	1	1	3	–1
Ovid MEDLINE to Scopus	14	21	4	259	0	1	12	27	–12
Ovid MEDLINE to PubMed	42	48	135	261	3	14	5	15	–2
Ovid MEDLINE to Embase	20	16	21	101	1	3	6	21	–5
Ovid MEDLINE to Cochrane	19	30	43	459	2	4	15	29	–13
Ovid MEDLINE to CINAHL	18	15	4	52	0	1	3	9	–3
Ovid MEDLINE to all databases	130	145	215	1150	2	8	8	21	–6
All translations to all databases	174	192	226	1207	1	7	6	18	–5

Abbreviation: PST-A=Polyglot Search Translator–assisted; SD=Standard deviation.

JMLA Journal of the Medical Library Association

Supplemental content to
J Med Libr Assoc. Apr;108(2):dx.doi.org/10.5195/jmla.2020.834
www.jmla.mlanet.org
© Clark, Sanders, Carter, Honeyman, Cleo, Auld, Booth, Condron, Dalais, Bateup, Linthwaite, May, Munn, Ramsay, Rickett, Rutter, Smith, Sondergeld, Wallin, Jones, Beller 2020

Table S7 Full list of number and percent of unique errors in search string translations

Error category	PST-A method translations (n=174)		Manual method translations (n=192)	
	n	(%)	n	(%)
Wrong wildcard or truncation syntax: recall*	30	(17%)	28	(15%)
Incorrect subject term conversion: recall	16	(9%)	11	(6%)
Not exploding a subject term: recall*	14	(8%)	15	(8%)
Missing or added wildcard or truncation: recall	12	(7%)	25	(13%)
Incorrect field syntax used: recall*	12	(7%)	7	(4%)
Incorrect search structure: recall*	11	(6%)	12	(6%)
Incorrect field chosen: precision*	10	(6%)	31	(16%)
Out of place characters inserted: recall*	10	(6%)	6	(3%)
Incorrect field chosen: recall*	9	(5%)	31	(16%)
Incorrect phrase translation: recall*	9	(5%)	9	(5%)
Missing subject term: recall	8	(5%)	16	(8%)
Spelling mistake in search: recall	8	(5%)	15	(8%)
Missing keyword term: recall	7	(4%)	25	(13%)
Incorrect adjacency syntax: recall*	7	(4%)	1	(1%)
Incorrect subject term conversion: precision	6	(3%)	2	(1%)
Exploding a subject term: precision*	5	(3%)	24	(13%)
Term truncated at wrong point: recall	5	(3%)	12	(6%)
Incorrect phrase translation: precision*	4	(2%)	25	(13%)
Incorrect adjacency used: precision	4	(2%)	9	(5%)

JMLA Journal of the Medical Library Association

SC
Supplemental content to
J Med Libr Assoc. Apr;108(2):dx.doi.org/10.5195/jmla.2020.834
www.jmla.mlanet.org
© Clark, Sanders, Carter, Honeyman, Cleo, Auld, Booth, Condron, Dalais, Bateup, Linthwaite, May, Munn, Ramsay, Rickett, Rutter, Smith, Sondergeld, Wallin, Jones, Beller 2020

Error category	PST-A method translations (n=174)		Manual method translations (n=192)	
	n	(%)	n	(%)
Not restricted to major subject term: precision*	3	(2%)	5	(3%)
Restricted to major subject term: recall*	3	(2%)	4	(2%)
Incorrect or missing Boolean operator: recall	3	(2%)	1	(1%)
Incorrect field syntax used: precision*	2	(1%)	1	(1%)
Extra components added: precision	2	(1%)	1	(1%)
Missing keyword term: precision	2	(1%)	0	(–)
Incorrect adjacency used: recall	1	(1%)	4	(2%)
Term truncated at wrong point: precision	1	(1%)	3	(2%)
Missing subject term: precision	1	(1%)	2	(1%)
Extra components added: recall	1	(1%)	0	(–)
Missing or added wildcard or truncation: precision	0	(–)	2	(1%)
Incorrect adjacency syntax: precision*	0	(–)	1	(1%)
Incorrect search structure: precision*	0	(–)	1	(1%)
Total	206		329	

Abbreviation: PST-A=Polyglot Search Translator–assisted; *=Error identified and fixed in the PST after trial completion.
