# Supplementary material for: Improving the translation of search strategies using the Polyglot Search Translator: a randomized controlled trial
Source: J Med Libr Assoc. 2020 Apr 1;108(2):195–207. doi: 10.5195/jmla.2020.834 (PMC7069833; doi:10.5195/jmla.2020.834)
Supplement: Appendix H [file jmla-108-195-s008.pdf]

JMLA Journal of the Medical Library Association

Supplemental content to
J Med Libr Assoc. Apr;108(2):dx.doi.org/10.5195/jmla.2020.834
www.jmla.mlanet.org
© Clark, Sanders, Carter, Honeyman, Cleo, Auld, Booth, Condron, Dalais, Bateup, Linthwaite, May, Munn, Ramsay, Rickett, Rutter, Smith, Sondergeld, Wallin, Jones, Beller 2020

Improving the translation of search strategies using the Polyglot Search Translator: a randomized controlled trial

Justin Michael Clark; Sharon Sanders; Matthew Carter; David Honeyman; Gina Cleo; Yvonne Auld; Debbie Booth; Patrick Condron; Christine Dalais; Sarah Bateup; Bronwyn Linthwaite; Nikki May; Jo Munn; Lindy Ramsay; Kirsty Rickett; Cameron Rutter; Angela Smith; Peter Sondergeld; Margie Wallin; Mark Jones; Elaine Beller

APPENDIX H

Table S8 Median percentage difference in the number of references retrieved relative to the number of references retrieved by the reference standard (with ranges)

Translation scenario	PST-A method		Manual method	
	Median % difference	Range	Median % difference	Range
PubMed to Web of Science	–1.47	(–87.3 to 600.0)	22.8	(–77.2 to 625.7)
PubMed to Scopus	768.3	(–95.2 to 18,700.0)	46.1	(0.8 to 28,000)
PubMed to Ovid MEDLINE	–12.9	(–66.7 to 82.4)	–17.3	(–57.8 to 160.2)
PubMed to Embase	–2.5	(–60.7 to 86.6)	–17.9	(–37.9 to 19.8)
PubMed to Cochrane	–1.1	(–100.0 to 486.9)	34.6	(–48.5 to 1,467.2)
PubMed to CINAHL	13.8	(–38.6 to 1,262.5)	43.0	(–4.6 to 300.0)
PubMed to all databases	–1.5	(–100.0 to 18,700)	2.1	(–77.2 to 28,000.0)
Ovid MEDLINE to Web of Science	11.7	(–75.9 to 596.7)	7.0	(–78.3 to 731.9)
Ovid MEDLINE to Scopus	22.5	(–41.2 to 2,012.5)	21.9	(–91.4 to 7,424.8)
Ovid MEDLINE to PubMed	2.4	(–98.8 to 753.1)	20.4	(–97.5 to 4,658.5)
Ovid MEDLINE to Embase	–23.1	(–99.6 to 7.2)	–12.4	(–89.7 to 13.7)
Ovid MEDLINE to Cochrane	2.4	(–100.0 to 39,716.7)	1.4	(–98.9 to 1,562.9)
Ovid MEDLINE to CINAHL	40.6	(–84.9 to 563.0)	104.0	(–36.6 to 2,984.7)

JMLA Journal of the Medical Library Association

Supplemental content to
J Med Libr Assoc. Apr;108(2):dx.doi.org/10.5195/jmla.2020.834
www.jmla.mlanet.org
© Clark, Sanders, Carter, Honeyman, Cleo, Auld, Booth, Condron, Dalais, Bateup, Linthwaite, May, Munn, Ramsay, Rickett, Rutter, Smith, Sondergeld, Wallin, Jones, Beller 2020

Translation scenario	PST-A method		Manual method	
	Median % difference	Range	Median % difference	Range
Ovid MEDLINE to all databases	3.0	(–100.0 to 39,716.7)	7.0	(–98.9 to 4,658.5)
All translations to all databases	1.7	(–100.0 to 39,716.7)	6.3	(–98.9 to 4,658.5)

Abbreviation: PST-A=Polyglot Search Translator–assisted.
